# Laying Anchor: Inserting Precision Health into a Public Health Genetics Policy Course

**DOI:** 10.3390/healthcare6030093

**Published:** 2018-08-03

**Authors:** Stephen M. Modell, Toby Citrin, Sharon L. R. Kardia

**Affiliations:** 1 Department of Health Management and Policy, University of Michigan School of Public Health, 1415 Washington Heights, Ann Arbor, MI 48109-2029, USA; tcitrin@umich.edu; 2 Department of Epidemiology, University of Michigan School of Public Health, M5174, SPH II, 1415 Washington Heights, Ann Arbor, MI 48109-2029, USA; skardia@umich.edu

**Keywords:** precision medicine, precision public health, education, genetics, cancer, cardiovascular disease, population health, race, health disparities, health policy

## Abstract

The United States Precision Medicine Initiative (PMI) was announced by then President Barack Obama in January 2015. It is a national effort designed to take into account genetic, environmental, and lifestyle differences in the development of individually tailored forms of treatment and prevention. This goal was implemented in March 2015 with the formation of an advisory committee working group to provide a framework for the proposed national research cohort of one million or more participants. The working group further held a public workshop on participant engagement and health equity, focusing on the design of an inclusive cohort, building public trust, and identifying active participant engagement features for the national cohort. Precision techniques offer medical and public health practitioners the opportunity to personally tailor preventive and therapeutic regimens based on informatics applied to large volume genotypic and phenotypic data. The PMI’s (*All of Us* Research Program’s) medical and public health promise, its balanced attention to technical and ethical issues, and its nuanced advisory structure made it a natural choice for inclusion in the University of Michigan course “Issues in Public Health Genetics” (HMP 517), offered each fall by the University’s School of Public Health. In 2015, the instructors included the PMI as the recurrent case study introduced at the beginning and referred to throughout the course, and as a class exercise allowing students to translate issues into policy. In 2016, an entire class session was devoted to precision medicine and precision public health. In this article, we examine the dialogues that transpired in these three course components, evaluate session impact on student ability to formulate PMI policy, and share our vision for next-generation courses dealing with precision health. *Methodology*: Class materials (class notes, oral exercise transcripts, class exercise written hand-ins) from the three course components were inspected and analyzed for issues and policy content. The purpose of the analysis was to assess the extent to which course components have enabled our students to formulate policy in the precision public health area. Analysis of student comments responding to questions posed during the initial case study comprised the initial or “pre-” categories. Analysis of student responses to the class exercise assignment, which included the same set of questions, formed the “post-” categories. Categories were validated by cross-comparison among the three authors, and inspected for frequency with which they appeared in student responses. Frequencies steered the selection of illustrative quotations, revealing the extent to which students were able to convert issue areas into actual policies. Lecture content and student comments in the precision health didactic session were inspected for degree to which they reinforced and extended the derived categories. *Results*: The case study inspection yielded four overarching categories: (1) assurance (access, equity, disparities); (2) participation (involvement, representativeness); (3) ethics (consent, privacy, benefit sharing); and (4) treatment of people (stigmatization, discrimination). Class exercise inspection and analysis yielded three additional categories: (5) financial; (6) educational; and (7) trust-building. The first three categories exceeded the others in terms of number of student mentions (8–14 vs. 4–6 mentions). Three other categories were considered and excluded because of infrequent mention. Students suggested several means of trust-building, including PMI personnel working with community leaders, stakeholder consultation, networking, and use of social media. Student representatives prioritized participant and research institution access to PMI information over commercial access. Multiple schemes were proposed for participant consent and return of results. Both pricing policy and Medicaid coverage were touched on. During the didactic session, students commented on the importance of provider training in precision health. Course evaluation highlighted the need for clarity on the organizations involved in the PMI, and leaving time for student-student interaction. *Conclusions*: While some student responses during the exercise were terse, an evolution was detectable over the three course components in student ability to suggest tangible policies and steps for implementation. Students also gained surety in presenting policy positions to a peer audience. Students came up with some very creative suggestions, such as use of an electronic platform to assure participant involvement in the disposition of their biological sample and personal health information, and alternate examples of ways to manage large volumes of data. An examination of socio-ethical issues and policies can strengthen student understanding of the directions the Precision Medicine Initiative is taking, and aid in training for the application of more varied precision medicine and public health techniques, such as tier 1 genetic testing and whole genome and exome sequencing. Future course development may reflect additional features of the ongoing *All of Us* Research Program, and further articulate precision public health approaches applying to populations as opposed to single individuals.

## 1. Introduction: The Precision Medicine Initiative—Its Expansion and Course Insertion

### 1.1. Course Structure and Prior Precision Health Components

The Precision Medicine Initiative (PMI) was created as a collaboration between the U.S. National Institutes of Health, health-related institutions and organizations, and the American public to research and develop individually tailored forms of therapy and prevention based on the amassing of genetic, environmental, and lifestyle data. The PMI has made news headlines and is now a solid part of the health-related literature, but its breadth and implications need translation to the professional educational level. In the pages to follow, we describe an innovative effort to include the PMI and precision health in a public health genetics policy course. “Issues in Public Health Genetics” (HMP 517) has now been taught at the University of Michigan School of Public Health for more than fifteen years. The instructors’ backgrounds are in law (Toby Citrin, J.D., chief instructor) and medicine and public health (Stephen M. Modell, M.D., M.S., co-instructor). The course is designed to acquaint students, typically of public health and genetic counseling, with the array of technical, ethical, and social issues facing professionals in the area of public health genetics, and with methods to convert issues into health policy [1]. We define policy as decisions made by public, private, professional, and community groups and organizations to affect behavior and direct resources. The policies focused on in the course range from the implementation of genetic screening programs to changes needing to take place at the state and federal levels to allow implementation and regulation of new genetic technologies to occur.

The “Issues in Public Health Genetics” semester consists of twenty-five one and a half hour long classroom sessions. The course as a whole is divided into four segments: A. “Policy Development” (nine sessions covering alternative methods of policymaking and their socio-ethical bases, from the fundamentals of the policymaking process to genetics law and legislation); B. “Individual, Community, and Professional Issues” (frameworks different professionals use in the design of genetic testing and screening programs, including consideration of race-ethnicity and health disparities—seven sessions); C. “Health Industry Issues” (issue areas and policy tools relating to genetics and the health industry, such as marketing issues, technology transfer, and consumer perspectives—five sessions); and D. “Looking Back and Looking Forward” (historical and cutting edge developments from eugenics to gene therapy—five sessions).

The sessions follow a seminar format, allowing considerable give-and-take and mutual discovery between its two instructors and students, as well as between the students themselves. Before class begins, the professors post on Canvas, the electronic curricular system, an average of three pertinent assigned readings, a number of supplementary readings, and a sheet with two to three questions to be discussed in class for the particular session. Typical questions ask how students would launch a program or policy, and how they would implement it. By the second session, a two- to three-page case study is circulated to the students (past examples: epigenetic research, biobanks, direct-to-consumer genetic testing). The case study for the semester presents the students with a larger number of socio-ethical and policy questions than a typical session, generally in the neighborhood of ten, which are discussed by session 3 in combination with an instructor presentation on policy frameworks. The case study is referred to recurrently throughout the course, and thus binds the course together thematically for the semester.

The one and a half hour sessions generally start out with 10 to 15 slides presented by the two instructors. Examples provided are brief, in comparison to the larger recurrent case study. Throughout, the intention is to move the students beyond just the scientific techniques being employed and the ethical issues they generate, to consider professional, institutional, and governmental policies and how they could be implemented. The goal of the course is to enable to students to formulate policy in the genetics area. To promote this goal, the instructors stop at various points in their presentation and ask for general thoughts, issues the students might see as important, and how an issue raised by students could be translated into policy. This formal presentation period, however, lasts no more than half the class period. The rest of the period is devoted to class discussion of the questions placed on Canvas for the session. This portion of every class allows a flow of questions, suggestions, and comments between students and instructors, with occasional probing questions offered by the instructors. Class composition, consisting of 10 to 20 students per year, remains constant throughout the semester. The students become familiar with each other and the course protocol fairly quickly. The first several class sessions are whole group, later evolving into alternating use of whole group and break-out group discussion, depending on the particular session. The break-outs are achieved either by dividing the classroom longitudinally, or allowing the students to self-assign themselves to one of three to four possible groups. Fifteen percent of a student’s grade is based on class participation and their follow-up postings in Canvas discussions. Thirty percent of the grade is based on two class exercises, one of which follows the theme of the recurrent case study. One of the class exercises involves individual student presentations; the other group presentations. Students self-assign themselves to roles for both varieties of class exercise format. The exercises are mostly occupied by the students presenting in response to a two to three-page class exercise sheet provided to them well in advance. The instructors also briefly introduce the exercise at the beginning, and conclude each individual or group presentation with a spontaneous question or two.

In terms of course content, the “Genetic Tools for Public Health Practice” session within segment B looks at various examples of genetic screening (prenatal, newborn, and adult, including use of family health history) and the ethical issues involved. The U.S. Centers for Disease Control and Prevention Office of Public Health Genomics (CDC-OPHG) has developed a framework of genetic conditions based on a three-tiered structure. Tier 1 conditions have techniques of genetic risk assessment that have been systematically validated [2]. We describe how screening newly diagnosed cases of colorectal cancer for Lynch syndrome, the most common heritable form of colorectal and endometrial cancer, can identify both index cases and their relatives requiring further attention. Low-density lipoprotein receptor (LDLR) mutation testing, and in some cases, APOB and PCSK9 testing, is called for in adults whose cholesterol measures have passed a critical threshold, and in children where family testing for familial hypercholesterolemia has identified a mutation. Genetic testing in these instances is criterion-based and aimed at the individual, bypassing testing of a wider clinical or general subpopulation (conventional testing for cholesterol lipoproteins has been advocated by some professional organizations for all children 21 years of age or younger). Tier 1 genetic testing for rare, high penetrance conditions, where either use of family history or genetic testing has been fully validated, is an important constituent of precision public health [3]. These forms of testing are not without controversy, such as whether testing should be “universal” among those at risk, availability of coverage, and privacy issues connected with contact of other family members. We bring these areas to the attention of our students so they can become acquainted with the considerations going into genetic testing implementation.

Each year, the syllabus is revised to reflect the latest genetic developments. In 2013, the American College of Medical Genetics and Genomics came out with policy recommendations on the reporting of incidental (secondary) findings in whole exome (WES) and whole genome (WGS) sequencing [4]. These forms of next-generation sequencing are especially valuable for the study of rare Mendelian conditions, and for identifying the genetic variants in all of a person’s genes (genome) and genetic coding regions (exome). Next generation sequencing has allowed clinicians to identify rare, private mutations, undetectable by standard genetic testing, in adult cancer and cardiovascular cases [5,6], and previously insoluble childhood cases involving conditions that would otherwise be fatal [7]. Serious mutations of mutual interest to the medical and public health communities that were detectable by sequencing include hereditary breast and ovarian cancer, Lynch syndrome (hereditary nonpolyposis colorectal cancer), hypertrophic cardiomyopathy, and long QT syndrome, a potentially fatal heart arrhythmia characterized by Q-T interval elongation on the electrocardiogram. In the clinical domain, whole genome and exome sequencing represent significant techniques in the burgeoning field of precision medicine, as the genes detected have allowed administration of life-saving personalized regimens. Use in the public health domain for newborn screening remains a contentious possibility, which is not yet realized [8,9]. Ethical issues involving right not-to-know, age of testing and reporting in pediatric cases, and “Who should decide?” arise [10,11]. In 2014, Wendy Uhlmann, a clinical associate professor and genetic counselor in internal medicine and human genetics at the University of Michigan, introduced the topic of disclosure of incidental findings in WGS into segment B of the course.

### 1.2. Advent of the U.S. Precision Medicine Initiative

In January 2015, then President Barack Obama announced the “Precision Medicine Initiative”, which was touted as “a bold new research effort to revolutionize how we improve health and treat disease” [12]. Efforts were underway to bring about new opportunities for individualized interventions through sponsored PMI research at major academic institutions and the establishment of a million person cohort for conducting this research. The seedbed for the initiative lay in the fruits of prior personalized medicine efforts, which had yielded now heavily used pharmacogenomic regimens such as Gleevec for chronic myelogenous leukemia and other tyrosine kinase inhibitors such as gefitinib for non-small-cell lung cancer [13]. The ongoing creation of new biobanks and genome-wide study consortia internationally, both public and private, were forerunners. Indeed, eight years prior the U.S. Health and Human Services Secretary’s Advisory Committee on Genetics, Health, and Society had issued a report on the feasibility of a large U.S. population cohort study of genes, environment, and disease [14]. The unique opportunity now taking place was that large amounts of genotypic information on rare and more common (single nucleotide polymorphism—SNP) variants could be collected. New targeted therapies could be developed based on the resources of an immense pooled repository of genetic and other health information, in the hopes that the outgoing discoveries could be applied in a highly personalized way in real time.

The President’s announcement inaugurated a string of events that proceeded at a fast clip as we were planning our course syllabus for fall 2015 and again for 2016. In March 2015, the U.S. National Institutes of Health (NIH) announced the formation of the Precision Medicine Initiative Working Group of the Advisory Committee to the (U.S. National Institutes of Health; NIH) Director [15]. In July, the working group held a public workshop on participant engagement and health equity as they related to the proposed national research cohort [16]. On 17 September (nine days after our course start-up for 2015), the PMI Working Group delivered to the Advisory Committee a report providing the framework for building a national research cohort of one million or more Americans [17].

The public health community was attuned to the start-up of the Precision Medicine Initiative. Muin Khoury of the U.S. Centers for Disease Control and Prevention Office of Public Health Genomics (CDC-OPHG) and colleagues published nine articles in the span of 2015–2016 touching on the relationship of precision medicine to public health and cancer screening [18,19,20,21]. In addition, in 2015, the *American Journal of Public Health* (AJPH) carried a major editorial on precision medicine and health disparities [22]. The equity concern has been an integral part of our course, as the public health code of ethics is deeply concerned with the health and empowerment of disenfranchised community members [23]. 

The leadership of the PMI has shown a great willingness to enroll as broad a swath of 1 million plus participants as possible, with inclusion of participants from varied racial-ethnic and socio-economic backgrounds. Its recently stated goal is to enroll 70 to 75% of the participants from individuals traditionally underrepresented in biomedical research [24]. The NYC Consortium, a PMI research center sub-awardee consisting of Columbia, Weill Cornell, New York–Presbyterian, and NYC Health + Hospitals/Harlem, for example, has a goal of “deliver[ing] equitable and culturally responsive care to the city’s most vulnerable populations” [25]. Its precision medicine program is applying an individualized approach to treating cancers; genetic diseases; and a broad range of other illnesses across an ethnically, culturally, and socioeconomically diverse population. In 2017, the PMI expanded its national network of provider organizations from the deep South to Wisconsin in the North, and from regional medical centers to local community health centers. Blue Cross Blue Shield and Walgreens are two of the organizations helping to enroll participants.

In segment A of the class, we have a “public deliberation” session that acquaints students with examples from a variety of settings of rational democratic deliberation—reasoned discussion on key values taking place in an atmosphere of mutual respect [26]. One such instance was the Oregon Health Experiment, “Oregon Health Priorities for the 1990s”, aimed at determining the health services citizens considered most important and, therefore, of highest priority for government funding [27]. A laudable number of local citizen forums, that is, nineteen, were held throughout the state, with 560 people completing the priorities survey, the results of which went to the Oregon legislature. A gender balance of 196 male and 358 female individuals participated. However, 56% of the participants worked in healthcare, and it has been observed that the preponderance of participants were white and well-educated. The PMI seems to have overcome this demographic limitation through vigorous efforts at expanding its enrollee provider network.

We felt it essential to integrate the Precision Medicine Initiative into the course, in a way that fulfills the public health concern with the health of the community and the rights and welfare of its members. Initially, we included the PMI in the course as a case study and class exercise (fall 2015), then integrated it as a full didactic session in fall 2016. This article reviews the three PMI educational components, with special attention paid to student involvement and the issues that were discussed and deliberated over. In the center is a brief qualitative analysis of student responses in one of those components—a class exercise. Our piece ends with a glimpse of future possibilities for inclusion of precision medicine and precision public health in the curricula experienced by budding health professionals.

## 2. Methods

Our analysis of the three Precision Medicine and Public Health course components resembles a “nested case-control design”, which situates a case-control study in the context of a cohort study [28]. Here, we nest a brief qualitative study of the student responses in the Precision Medicine and Public Health case study and class exercise between the above narrative description of previous course elements touching on precision health, and an examination of the precision medicine and public health topics covered and student comments rendered in the Precision Medicine Initiative didactic session. The two aims of this analysis are the following: (1) show how precision health, the PMI in particular, can be incorporated into a public health genetics policy course; and (2) assess the extent to which this inclusion has allowed our students to formulate policy in the precision public health area.

Knowing that precision public health was a new area with lasting implications, the course instructors took extensive notes on student–instructor and student–student dialogue during the three course components. Verbatim records of student presentations are kept during class exercises to enable accurated grading. The instructors have inspected and analyzed for issues and policy content relevant data for the three Precision Medicine Initiative-related sessions: (1) class notes containing what each participant said—for all three sessions; (2) student hand-ins for the class exercise; and (3) the topical areas covered in the class materials of the didactic session. The didactic session topical areas were inspected, following analysis of the case study and class exercise, for content that reinforces and extends the themes elicited by the case study and class exercise, and illustrative student comments.

In the analysis, student responses for the case study and class exercise, both oral and written, were categorized into thematic areas. The pre-categories were formed by assorting themes in the ten shared case study/class exercise questions (page 3 of the case study and class exercise hand-outs) into issue and policy areas. The post-categories were formed by manually labeling sentences in the case study and class exercise student responses for major themes. Categories were cross-checked by the three authors. The authors recorded frequency of mentions in the pre-and post-categories for the following: (1) case study student responses; (2) in-class exercise student responses; and (3) class exercise hand-ins, which were used to select exemplary quotations within the major categories. In keeping with the course policy orientation, we have analyzed student responses to determine our students’ ability to formulate issue areas into policy, that is, to satisfy aim 2, rather than to systematically explore the various categories of responses. We also include student comments on the Precision Medicine and Public Health class exercise, collected as part of the overall end-of-semester course evaluation, to show whether students felt the class exercise was useful and what changes could be made in the future. The evaluation did not include questions on the case study and didactic session.

In writing this piece, the authors have inspected the precision medicine and precision public health technical, program-related, and socio-ethical literature we collected at the time of the 2015/2016 classes, and supplemented this inspection with additional current PubMed and NIH website searches. The PMI has evolved into the NIH *All of Us* Research Program, which began beta testing in June 2017 and had a full national roll-out of the cohort-based program and extensive provider network in Spring 2018. This article is written from the standpoint of what has taken place in the national program as of June 2018.

## 3. Results

### 3.1. Course Moorings: Findings from the Precision Medicine and Public Health Case Study

“Issues in Public Health Genetics” uses the case study approach in session 3 to ground students in principles of policymaking, and the different bodies and phases involved [29]. For the fall 2015 Case Study in Precision Medicine and Public Health, students were provided in advance with a two-page description of the PMI, 10 ethical-legal-social implications (ELSI)/policy-related questions, a policy types sheet, two assigned and two supplementary readings on precision medicine and public health and the PMI, and a number of general and genomics-specific policy readings. The case study begins with former U.S. President Barack Obama’s announcement of the Precision Medicine Initiative. The Initiative announcement envisioned a new era of medicine wedding together novel research, technology, and policies aimed at the development of innovative treatments [12]. The instructor-provided description distinguishes PMI near-term and long-term goals. In the near-term, the PMI was directed at supporting clinical trials in partnership with pharmaceutical companies to test combinations of targeted therapies. The therapies being tested together with enabling diagnostic tests have focused especially on cancer and tumor molecular signatures [30]. The PMI’s longer term goal, now proceeding at a brisk pace, is the creation of a national cohort study of one million or more Americans. Students read the ambitious description of the million person enterprise as it originally appeared on the National Institutes of Health website:

“Each voluntary participant will share their genomic information and biological specimens. This information, along with important clinical data from electronic health records, such as laboratory test results and magnetic resonance imaging (MRI) scans, and lifestyle data, such as calorie consumption and environmental exposures tracked through mobile health devices, will help researchers understand how genomic variations and other health factors affect the development of disease. Through the consent process, participants will control how the information is used in research and shared. As active participants, they also will have access to their own health data, as well as research using their data, to help inform their own health decisions” [31].

One can see that the PMI’s goals are laden with both immense potential and equally large design challenges. We capitalize on the challenges to allow our students to analytically move through the ELSI and policy considerations necessary for accomplishing the PMI’s stated goals. We ask our students to consider the 10 questions posted on the course Canvas website before class and to be prepared to discuss in class relate to the following: (1) assurance of services (access to data and results, maintaining equity, minimizing health disparities); (2) participation in the PMI (involvement, representativeness); (3) ethical issues (consent, privacy, and benefit sharing); and (4) treatment of people (stigmatization, discrimination). These four overarching “pre-” categories follow from a direct inspection of the questions in Table 1, as performed by the three authors.

Following instructor presentation of policymaking frameworks, the case study discussion began with give-and-take between instructors and students to lay-out the process of realizing the PMI’s goals. The class envisioned the process as containing four steps: (1) announcement of the PMI’s near-term and longer-term goals (which students had read about); (2) incorporation of these plans into the Congressional budget; (3) NIH director to map-out the division of money for the PMI and its various needs; and (4) formation of an advisory group, with subgroups as needed and a stakeholder group (suggested by the chief instructor, who was privy to their activities in the months before class began). The instructors noticed that the students were not accustomed to connecting policymaking steps in this manner, but expected them to gain familiarity with this mode of thinking as the semester proceeded.

The students were then asked for those issues they found most pressing. Student responses to the case study fell into two of the four preliminary categories above—participation (2 responses) and ethics (privacy—3 responses; consent—2 responses). One student felt consent was a priority, given that it would be needed for use of the samples. The student mentioned that consent was a part of the U.S. Office for Human Research Protections (OHRP) regulations. She was asked for examples, and offered the ongoing debates about dried blood spots being stored by states as instances where government programs were likely to benefit from more structured consent policy. In the case of the PMI, an advisory commission could start with recommendations, and then government agencies (Secretary of Health and Human Services, NIH, or state health departments, she suggested) could firm up the consent policies. The student proposed researchers and public groups working together early on to fashion policy.

A second student felt that the need for privacy safeguards was paramount. He remarked, “These have changed the last ten years with the Internet. Does the federal government have the capacity to handle million person data?” This class member took a practical approach to the problem—“Hire the right people; pull lessons from the U.S. census”. On being asked whether a federal agency could be granted authority over the privacy of samples and personal health information, he suggested that responsible private vendors could be used. Students and instructors then explored whether and how the data could be de-identified.

Towards the end of the discussion, the instructors asked the class how diversity of the national cohort could be assured. The conversation over this topic needed more instructor prompting than previously, suggesting that the students, either at the undergraduate or graduate level, had received more exposure to ethical than social dilemma problem-solving. Representativeness of the cohort would not only make it more technically useful, but also improve trust among groups that had been either ignored or mistreated by past biomedical research. Students were further asked to consider, “Who gets the benefits?” Would private sector biotech be the primary beneficiary? Should consumers’ interests not be considered foremost? The class was reminded that public health was not well represented in the advisory groups convened by NIH, and that the public health community was a stakeholder as well.

All of these lines of inquiry elicited fairly detailed and pragmatic student responses, more so than the instructors had expected. However, it was clear, as demonstrated in the questioning about participant representativeness questioning, that the discussion could be taken further after additional student exploration. The student responses had also been somewhat scattershot in terms of the steps in policymaking that would be needed. The case presentation, therefore, provided a scaffolding for a more incisive precision medicine and public health exercise that would appear further down the road in the course. The questions students grappled with would be revisited. Introduction of the case study at this early point in the course also allowed periodic reference to precision medicine and public health considerations throughout the course.

### 3.2. Setting Sail: Findings from the Precision Medicine and Public Health Class Exercise

The questions posed to our students in the session 3 case study on precision medicine and public health provided an initial exposure to the relevant issues and the chance for students to loosely consider positions on the issues leading to policies. The session 16 precision medicine and public health class exercise took place slightly after the course midpoint. Students would now have the opportunity to offer individual recommendations and to defend them. At this stage in the course, students had been exposed to sessions on the policymaking process, genetics legislation, public deliberation, ethical perspectives, issues of race and ethnicity, and tools for public health practice. Although not always the case in the years we have taught the course, we designed the narrative to be usable in both the case study and exercise sessions in order to maintain student familiarity with it. Other than the specific assignment, the descriptive narrative and 10 questions were identical in both the case study and class exercise hand-outs, the latter being distributed several weeks in advance. In addition, by the time of the class exercise, mention of the Precision Medicine Initiative (PMI) had been made recurrently throughout the prior sessions.

The individual class exercise added a context to the questions posed in the case study. In the class exercise scenario contained in the advance hand-out, students learned that the NIH had established a working group on precision medicine as a component of the NIH advisory committee to the Director. The actual PMI working group had delivered its culminating report on a framework for a one million plus research cohort to the advisory committee to the NIH Director four weeks before the class exercise. The student involvement in decision-making thus paralleled what was taking place in the real world. In the hand-out, the working group on precision medicine was to convene a meeting to hear testimony from invited stakeholders recommending policies to address ELSI aspects of the Precision Medicine Initiative. Students were asked to choose in advance from a list of 19 possible professional, industry-based, and civic organizations. One student reminded us in the end-of-course evaluation that “an overview of what the organizations are beforehand would save time for additional research on the actual policy issues”. This suggestion could be honored by supplying a one to two sentence description plus a web site for each organization. The supplementary readings we place on Canvas are broken down by organization category, thus reflect different stances that might be taken.

Before the class session, student stakeholders were required to submit a one to two-page memorandum describing their organization’s recommendations (the written exercise) online. During the in-class portion, each stakeholder was allotted 10 min to present their positions on relevant issues, and respond to one to two professor (advisory committee “chair” and “co-chair”) questions (the in-class exercise). Students self-assigned themselves to represent the following organizations: CDC’s Office of Public Health Genomics, American Society of Human Genetics (ASHG), National Society of Genetic Counselors (NSGC), Pharmaceutical Research and Manufacturers of America (PhRMA), Genetic Alliance, Council for Responsible Genetics, and the National Urban League. One student commented in the end-of-semester course evaluation, “I think it would be good for a student to perhaps be expected to take on both a governing/regulatory body and an advising group in order to see both roles”. While time constraints do not permit a student’s adopting two roles, we have added more time for open discussion, sometimes up to 15 min, in past class exercises. Dawning multiple perspectives for purposes of open discussion is a valuable suggestion, and might grant students a chance to voice positions that most represent how they personally feel. This conclusion is backed up by a second student’s evaluation comment: “I think maybe a shorter presentation (5 min) and more in character discussion would have been interesting”.

Assurance (14 oral responses; 13 written responses), participation (13 oral; 6 written), and ethics (26 oral—11 consent, 6 privacy, 9 benefit sharing; 19 written—8 consent, 6 privacy, 5 benefit sharing) were the three most frequent categories of student responses in both the oral and written exercise. New “post-” categories of responses also appeared, which we added to the four “pre-” categories from the case study: financial considerations, education, and trust-building. Treatment of people (stigmatization, discrimination), an earlier category, fell midway among these new categories in terms of frequency of mention; financial and educational considerations only received four to six responses, respectively. Low number of mentions in student responses prompted eventual exclusion of three contemplated categories: ethics-ownership, regulation, and translation.

In attending to the student responses, the chair and co-chair were checking to make sure that students had an appreciation of the advancements and challenges posed by the PMI, and that their proposals would positively and not negatively impact the participants according to the role they adopted. A 2018 report by Data & Society, *Fairness in Precision Medicine*, notes that large-scale precision medicine research studies, such as the *All of Us* Research Program, Project Baseline, and New York University’s Human Project, have made strides in prioritizing a diverse enrollment, a major step towards ensuring that benefits could be equitably distributed [32]. Data & Society conducted interviews with 21 individuals. The resultant report “stressed that varying sources of data would need to be used, but that harnessing these different sources would require tackling similar [historical and analytical] concerns regarding the potential for bias” (p. 24). The potential for sampling bias, due to the history of biomedical research studies focusing on volunteers of European descent, was voiced by our National Urban League student representative. The Genetic Alliance representative in our class exercise proposed: “We must connect and engage with community and advocacy groups, and then reach out to the public with the help of those groups and their web sites and means of communication”. These comments confirmed to us that our students had an appropriate level of attention for group hopes and concerns, which goes beyond simply being focused on the program or intervention itself. The students suggested the use of social media, such as Facebook and Twitter, as ways to create diverse involvement (Table 2). A means of avoiding recruitment bias is to allow the participants themselves to tap into the study description and self-enroll, rather than being enrolled according to fixed quotas that the study professionals have arranged.

The U.S. Precision Medicine Initiative initiated by former President Obama in 2015 has hoped to avoid the fate of the National Children’s Study (NCS), which planned to track 100,000 children from birth to adulthood [33]. This effort entailed a longitudinal study examining the influences of a range of factors, from chemical to psychosocial, on child development and health. Among the reasons for the study’s termination were a cumbersome recruitment plan, based on enrolling pregnant women by knocking on doors in a random sample of about 100 counties [34]. With a surge in costs, NIH dropped the 40 NCS sites at academic institutions, and transferred management of enrollees to several large contractors. More socially-based means can be used to propel recruitment, along the lines of the plans proposed by our students and through the type of network expansion being undertaken by the *All of Us* Research Program.

Success in enrollment and in the conduct of a research study is dependent on the trust of the participants. The Genetic Alliance student representative felt that operating through pre-established channels within community organizations, which requires a period of familiarization between public constituents and the research team, is a valuable way of engaging in trust building. In his oral presentation, the Pharmaceutical Research and Manufacturers of America (PhRMA) student representative suggested a “trust of stakeholders—public, provider, and industry”. The trust concept, consisting of an umbrella organization charged with fiduciary responsibility for seeing to the best interests of the participants, was first proposed by Howard University in 2003 for a biobank to investigate diseases that predominantly affect African Americans. Samples housed in the Genomic Research in African Diaspora (GRAD) biobank were to be managed by a private company with a fiduciary duty to the enrollees, the First Genetic Trust in Chicago [35]. The Michigan BioTrust for Health was created to enable research on the four million dried blood spots stored from newborn screening by the state of Michigan before 2010 [36]. A combination of retroactive opt-out and proactive informed consent policies have enabled the biobank to go forward with investigations as far ranging as epigenetic exposure and childhood brain tumor risk, to lab-on-a-chip for newborn metabolic disorders [37]. The Michigan BioTrust is overseen by both a scientific advisory board, and a community values advisory board. The latter provides advice on what types of research are ethically acceptable, and methods for engaging and informing the public. Similarly, the *All of Us* Research Program’s advisory panel and its working group of the advisory committee to the NIH Director formed to provide external oversight and expert advice on the program’s goals and operations. The July 2015 open workshop allowed outside public representation on the design of an inclusive cohort, building and sustaining public trust, and active and effective participant engagement [6]. Our National Society of Genetic Counselors (NSGC) student representative stated that, “Researchers and participants being equally involved would establish trust”. The ideal, as expressed by our students, would be an ongoing collaboration between experts and lay people. The 2015 open workshop and the continued presence of advocacy leaders on the program advisory committees are partial reflections of this shared student suggestion.

We were surprised that the number of ethics-related comments during the oral presentations exceeded the number of stigmatization/discrimination comments by a ratio of 5:1. This imbalance could be due to student’s previous exposure to bioethics, because as undergraduates, they were eying health fields, but it also serves as a wake-up call to medical and public health educators charged with communicating the implications of precision tools. The NSGC student representative cited the need to ensure ethical directions for the research so as to avoid discrimination. The student acknowledged the existence of the Genetic Information Nondiscrimination Act (GINA), at the same time recognizing its being limited to health insurance and not covering life or disability insurance. Considering the case of BiDil, which its manufacturer NitroMed, after a retrospective revisiting of the data, decided to market exclusively for heart failure in African Americans, forgoing other groups in its prescribing [38], the concern over discrimination is justified. Many authorities view BiDil as a forerunner to race-based pharmacogenomics. In an effort to give people control over the uses to which donated biological samples and personal health information were being put, two of our groups recommended utilization of the “platform for engaging everyone responsibly” (PEER) system. PEER is the product of a collaboration between Genetic Alliance and Private Access [39]. The system allows the health consumer to decide which types of research and research organizations will have access to their biological samples and personal information. Instructions can be changed at any time, making PEER’s operation dynamic through time. The PEER system is quite reasonable for applying to a moderate size biobank, such as the Genetic Alliance’s registry and biobank devoted to research on Pseudoxanthoma Elasticum, but can it serve a one million plus cohort? Because the *All of Us* Research Program cohort and provider networks are so immense, application of PEER might be too unwieldy. It should be noted, however, that *All of Us* Research Program research center sub-awardees have as their goal the examination of samples and health data on a much more limited scale. The Illinois Precision Medicine Consortium has launched a longitudinal research cohort of 125,000 participants, and the University of Michigan’s “Michigan Genome Initiative” has accumulated genomic and electronic health record data on 35,000 plus non-emergency surgical patients (The University of Michigan supplies tools and methodology to the *All of Us* Research Program’s Data and Research Support Center; its overall precision health activities are part of an institutional initiative). It would be feasible to either employ PEER at the sub-awardee level, or have participant input in an advisory sense help in establishing community trust concerning the directions the research is taking.

The ethics behind the million person cohort are based on maximization of benefit and avoiding maleficence-untoward, detrimental consequences. Our students portrayed benefit to research cohort participants as falling under two headings: (1) information to be received back by participants; and (2) treatments that might ultimately benefit participants or participant groups. Several student representatives mentioned the importance of new pharmacogenomics regimens being available to individuals of diverse socio-economic backgrounds. One representative stated in response to instructor questioning that the government should be responsible for payment of drugs, a suggestion that was simply too terse. We agree with a course evaluation comment asking us to “Encourage students to take concrete positions on issues”. Part of this role is fulfilled by asking the student to clarify their response during the question and answer session. Other students suggested institutional policies that would avoid excluding individuals because of prohibitory prices, and more specific governmental policies, such as continued expansion of Medicaid, to support cost-effective implementation of precision medicine. The National Urban League student representative suggested local groups, networks, and Genetic Alliance associated advocacy organizations could monitor and investigate potential health disparities. We look for student awareness of the need to include communities and community-based organizations in the research process, and commended this idea.

Comments regarding access to information from the PMI addressed both consumer and non-consumer needs. The PhRMA student representative felt that PMI information should be available only for research purposes, to qualified individuals from academia, government, and industry. Lifestyle information was viewed as more sensitive, with one group suggesting it should be released judiciously through “controlled channels”. While this point was well taken, we felt the distinction between sharing of genotypic and lifestyle plus phenotypic data, and their means of release by NIH, could have been further articulated. Our advocacy group student representatives endorsed open access, which would also allow participants to gain access to PMI information. A distinction needs to be made between accessing general information from the PMI, and one’s own information. In her oral presentation, the NSGC student representative cited a concern with non-return of results, and indicated that “98% of participants want their information back”. A large number of people surveyed by the Foundation for the National Institutes of Health indicated they would be more likely to participate knowing their health information would be returned [17]. The Center for Responsible Genetics (CRG) student representative discussed a “reverse identification” scheme for linking participant scientific and medical historical data back together to enable such personalized disclosure. The National Society of Genetic Counselors made a very insightful connection with results returned to individuals by direct-to-consumer genetic testing companies; adequate counseling resources would need to be in place.

The ability to report back to the patient, particularly after his or her biospecimen has been analyzed and compared with other comparables within a precision medicine database, is a real-time goal of precision medicine. Leaders such as Francis Collins of NIH and Muin Khoury of CDC have discussed such an ambition for diabetes treatment and smoking cessation [21,30]. In dialogues on the fruits of the Human Genome Project with the African American and Latino participants in our *Communities of Color and Genetics Policy* Project, we found that participants were interested in both maintaining privacy of results, and receiving their own genetic testing results back, even if it meant not anonymizing their sample [40]. In part, the reason against disclosure of individual results is ethical—results at the research stage are uncertain. This leaning differs from medical practice once a given diagnostic test has entered the medical mainstream after due validation. In addition, the re-linking of human subjects’ data, which no longer involves preliminary data but now research results, would be administratively impractical on a widespread scale. The CDC-OPHG does describe a process of aggregate result bidirectional reporting between state disease registries, for example, in the cancer field, and those institutions that contributed data to the registry [41]. Such information could be highly useful to providers and their patients for those groups being seen at a particular healthcare site. Accordingly, the Genetic Alliance student representative advocated public open access for articles published using million person cohort data.

Among our groups, PhRMA was the most optimistic in terms of the pay-off of the Precision Medicine Initiative. In addition to its student representative’s suggestion that the PMI’s goals could be maximized by allowing data to be shared with a variety of research stakeholders, he felt the “broadest possible consent” (blanket consent) should be obtained from participants. This viewpoint was counterbalanced by the perspective of the American Society of Human Genetics (ASHG) student representative, who wrote that any benefits of the publicly available data should be used “to benefit the overall health of the public”, a view that deemphasized institutional benefits. The Council for Responsible Genetics student representative was concerned about biobanks sharing information with corporations and the sale of personalized information by 23 and Me, implying that potential benefit and risk must both be considered when lifting confidentiality. The PhRMA and professional/advocacy viewpoints both had the health consumer in mind, but the circle of benefit was prioritized differently. The exercise was intended to draw out a spectrum or polarity of responses to the ethical, legal, and social issues inherent in precision medicine and public health. It appeared this interchange accomplished our purpose. We received a student course evaluation comment that “I know time is a constraint, but I was ready to have a discussion, in the role I was in, with the other stakeholders, and to try to hash out a policy. I wished there had been time for that”. Occasionally we have asked students or groups with likely contrasting positions to present sequentially, and incorporated a point raised by one group into a question aimed at the other group. Another technique is to allow a student to ask the second or third question during an individual’s or group’s question and answer session. Often, the question is based on a differing position. The time after presentations generally allows for student give-and take, but not for more thoroughgoing consensus building. Afterwards, class conversion via the electronic Canvas Discussion helps to fill this need.

### 3.3. Charting a Course: Findings from the Precision Medicine and Public Health Class Didactic Session

Upon inspecting the student responses within the 2015 case study and class exercise, we felt that our attendees should be provided with a more distinct definition of precision public health, as the PMI tends to focus on amelioration of the later stages of disease. In fact, the distinction between prevention-oriented and treatment-oriented precision techniques was a question we had asked our students, with one replying that where a condition lies along a “range of risk” could help to differentiate the model to adopt. In addition, several of our students in the exercise issued comments on the need to guarantee equity in the fruits of precision medicine, which strengthened our belief that a didactic class session emphasizing this topic should added. Session 12 in the fall 2016 curriculum occupied this intellectual space.

A Precision Public Health Summit was held on the 6–7 June 2016 at the University of California San Francisco. The Summit’s aim identified two major aspects of precision public health: “to look at ways that vast amounts of data can be used to benefit the health and well-being of larger populations, as well as to decrease health disparities” [42]. In the class, we present four characteristics molding precision health into the public health context: by utilizing (1) an ecological model of health; (2) principles of population screening; (3) evidence-based decision-making; and (4) public health policy and practice [21]. The U.S. National Academy of Medicine’s 2003 report *The Future of the Public’s Health in the 21st Century* defines an ecological model as one based “on understanding the ecology of health and the interconnectedness of the biological, behavioral, physical, and socio-environmental domains” [43,44]. The last domain introduces consideration of equity and class in connection with health.

The National Urban League student representative during the 2015 class exercise stated her support of the social determinants of health model, which emulates a public health ecological framework, and felt it should be a part of professional education. These models provide a rationale, when translated into effectiveness research, for targeting disparities in marginalized populations. She stated, “We have reservations about the huge costs of the Precision Medicine Initiative taking away resources and public interest from other policies that would be more impactful on these [urban] populations”. The student cited the *National Healthcare Quality and Disparities Report* indicating that across a range of measures of healthcare access, Latinos received equivalent care to whites in only 17% of healthcare measures [45]. African Americans and Latinos received poorer quality care than whites on 73% and 77%, respectively, of those measures. As we tell our students, improvements in health often demand more than medical attention. Ronald Bayer and Sandro Galea, whose writings on precision medicine and social justice we include in the course, cite the findings of the British Civil Service’s Whitehall Study. This investigation found that even when healthcare services were provided as a matter of right and the cost of care was no longer a barrier to treatment, a marked social gradient persisted [46]. A substantial proportion of the population fared poorly on health indicators. Bayer and Galea view targeting of public health efforts as optimal when they are broad-based and cross-sectoral. These considerations do not mitigate the value of the PMI, but they do indicate that for full effect, targeting will need to go beyond the individual level and take into account social determinants.

We asked the students in our class session, “Will precision medicine increase or decrease health disparities?” Part of the answer to minimizing disparities was addressed during the exercise by one student’s suggestion to make sure an adequate number of racial-ethnic minorities are recruited into studies and study centers being awarded by the NIH, especially when conditions disproportionately suffered by minorities, such as prostate cancer and asthma, are being investigated. Other healthcare reform era solutions, such as expanded coverage under the Affordable Care Act, are important. The outcomes of the *All of Us* Research Program will not always exist at the research stage. Targeted interventions will need to be paid for. To be avoided is “a high-tech personalized approach that [is] only available to those with means or platinum/Cadillac insurance plans” [42]. Our students brought up the wise point that in the case of regimens going beyond simply taking a pill at home, assurance of basic transportation to the health center through whatever arrangements can be made is fundamentally important.

Built into the original description of the PMI are means of engaging potentially marginalized populations: “The Obama Administration will forge strong partnerships with existing research cohorts, patient groups, and the private sector to develop the infrastructure that will be needed” [31]. Of course, it is to be hoped that the White House and the President’s Secretary of Health and Human Services will continue to support the PMI, regardless of which administration is in office. We cite the value of incentives, such as giving participants wearable health monitoring technology, and providing an interactive web site for people [47]. Like many biobanks, the Norwegian Nord-Trondelag Health (HUNT) Study biobank makes commercial use of its human biological samples, but an upfront public contribution fee from each project and financial return to the research and health communities are also fixtures in its sample management strategy [48]. Involving representatives of the research participants in governance is an important ingredient in furthering buy-in, and has taken place to some extent within the PMI working group and advisory structure. These representatives may hail from an advocacy group, or be members of a board, or even individuals from industry or academe, so long as they can serve as and are viewed as trusted representatives.

The last big issue that we entertain with our students, a logistical one, is the vast amount of information that will pour out of the PMI. Recall that the plan is to recruit more than one million Americans, and to capture genotypic, phenotypic, lifestyle, and environmental information on each. Whole-genome sequencing alone produces an immense amount of information. The question we naturally pose to our students is, “How can we deal adequately with the flood of information given the lack of time or expertise to interpret and apply the knowledge?” One instructor suggestion was to be sure the information trickling down from the Initiative is valid and not subject to misinterpretation. An astute pupil brought to the class’s attention the point that The Cancer Genome Atlas (TCGA), a collaboration between the National Cancer Institute and the National Human Genome Research Institute, could serve as an example. TCGA has a biospecimen core resource (BCR) that serves as the interface between the TCGA program and the various tissue source sites collecting tumor samples [49]. The BCR also ensures that human subjects protections and guidelines are adhered to at each of the collection sites. However, it is also the case that the TCGA program office does not provide to investigators help with bioinformatics or guidance on research projects using the data. We noted a student comment that the best way to ensure that precision medicine and public health do not widen health disparities would be to enable adequate provider training of and education of the involved public. Suitable expertise must exist at the clinician level, which will likely be the state of affairs for information flowing out of the PMI, too. The didactic session provided a useful capstone to the previous year’s precision public health activities, translating the major issues in consolidated form for our students.

### 3.4. Final Arrival: Summary of Findings for the Three Course Components

For the most part, we would give our students a positive rating in terms of their responses to instructor questions and thoughtfulness during class discussion. It was gratifying to hear their policy suggestions both for the process of policymaking (e.g., work with community leaders, conduct stakeholder consultations, engage IRBs to establish consent policy) and its substance (dynamic consent, data de-identification and anonymization, expanded payment policies). In so doing, the students came up with some very creative suggestions, such as use of the PEER electronic platform to assure participant involvement in the disposition of their biological sample and personal health information. We also heard a lot of timely responses involving research program-participant collaboration, and a suggestion for using social media to disseminate awareness of the program, which made us wish our students could have been there at the NIH public workshop. Students quite appropriately articulated the need for attention to consent and privacy policies. We did detect at the beginning of the course more student competence in solving ethical dilemmas than reasoning-out steps for implementing policies and narrowing social gaps in proposed programs, such as assuring representativeness. While some students gave overly concise replies to “chair” and “co-chair” queries during the exercise, for the most part, responses were practically grounded and reflected useful policy measures. Student weaknesses, including not knowing how to concisely articulate points to an audience under a fixed time limit, seemed diminished by the end of the exercise session. In terms of evaluation of the Precision Medicine and Public Health exercise by students who returned our internal questionnaire, five students rated it as very valuable (compared to four for the second, CRISPR class exercise); one rated it as somewhat valuable; and one circled “N/A”. The students in our class, largely health science-oriented, often broach in our final class debriefing that they do not feel completely at home in public health policymaking, but admit they have gained useful familiarity with the process, and that the class exercises have helped. The precision medicine and public health exercise furthered the course’s policymaking goal and motivated the students to want to collaborate with each other in designing policy. A limitation of the analysis we have performed is that because of its retrospective nature, we lack the capacity to evaluate student perceptions of the case study and didactic session.

## 4. Conclusions: A Look to the Horizon of Precision Public Health Education

In the didactic session, we have one slide with the straightforward title “Training Necessary”. Certainly, one solution for dealing with the plethora of information stemming from the Precision Medicine Initiative is to train the clinicians and young public health investigators who will feed the results back into their own research, and the medical and public health practitioners who will be using the information for their patients and community members. Mirnezami and colleagues foresaw the need for new training paradigms for doctors, “who will benefit from a deeper, more holistic view of illness integrating traditional pathophysiology-based models with emerging molecular mechanisms” [50]. This training requirement is engendered both by the new gene-disease associations that will be detected, and the volume of information the national cohort will generate. Though the educational level at which the training must take place may be uncertain, its necessity becomes manifest well before the time the provider is ready to apply a precision medicine intervention to his or her patient.

Much of the medical view of the PMI is pharmacogenomic, albeit with greatly enhanced informational and real-time capabilities. In May 2018, the *All of Us* Research Program announced funds to attain whole-genome sequencing of 200,000 people per year [24]. Whole-genome and exome sequencing can be of great utility in identifying new pharmacogenomic targets and solutions for medically refractory cases, particularly when big data aids the search [51,52]. These techniques are beginning to make their way into medical, other graduate level, and genetic counseling education [53]. A postgraduate program in clinical pharmacology and pharmacogenomics at the University of Chicago offers training in personalized therapeutics, and includes the ethics and economics of pharmacogenomics, as well as limited service aboard an IRB [54]. Web resources also exist to support practitioner utilization of pharmacogenomic information to guide drug therapy [55]. Genetic epidemiology courses in schools of public health include WGS and WES as research techniques. Public health practitioners are currently most likely to encounter WGS and WES in the epidemiologic tracking of virulent pathogens, which requires professional training [3]. Use in newborn screening remains under investigation. Our study suggests that training in these techniques can also benefit from an awareness of costs and accessibility by different consumer groups.

As our course lays out, public health practitioners are especially concerned with the health of the family, community, and population. Muin Khoury and Sandro Galea write that to-date, “substantial evidence indicates that a genetically targeted approach to health has demonstrated a population [not just an individual] health benefit” [18]. The example they supply is evidence-based tier 1 (tests fully validated) genomic interventions, which includes both index case and family cascade testing. Tier 1 genetic testing is already a solid topic in public health genetics courses like ours. The CDC has made available tool kits containing informational and technical resources for the implementation of tier 1 genetic testing [41].

A second precision approach medicine and public health are likely to adopt as the PMI proceeds is the use of polygenic risk scores, which incorporate genetic variants of both small and large effect size. Researchers at the University of Michigan School of Public Health and the Fred Hutchinson Cancer Research Center in Seattle, WA have combined risk information from 19 lifestyle and environmental factors and 63 genetic variants (“E-scores” and “G-scores”) with family history to yield an overall risk score for colorectal cancer [56]. Medical investigators in the United States and Canada have employed polygenic risk scores in the identification of individuals with a high burden of subclinical coronary atherosclerosis, and for genotypic analysis of individuals with extreme levels of high-density lipoprotein cholesterol [57,58]. Development of such scoring systems is likely to accelerate as further genotype-phenotype associations emerge from the *All of Us* Research Program and investigators connected with it. Training in genome-wide association studies is a major part of public health epidemiology coursework here at University of Michigan; the topic of “phenome-wide association studies” will follow as these studies increase in number [59]. Both techniques are used in the formation of risk scores. The American Public Health Association policy statement on genetic and genomic literacy indicates that provider training needs to include familiarity with risk stratification and its proper interpretation, and an awareness of circumstances for resorting to lifestyle modification versus pharmacologic measures [60].

The two approaches—(1) tier 1 genetic testing, and use of WGS and WES; and (2) use of polygenic risk scores—are basically the major genetic mutation and SNP versions of targeted interventions for at-risk populations. The use of such interventions requires education, ideally starting at the graduate level and extending to workforce training [60]. More will be asked of the patient or health consumer than in prior forms of testing. Education for future practitioners should contain elements of consent, confidentiality and data sharing, group sensitivity to informatics-based testing, costs, and coverage. As our students demonstrated, absorbing material related to these topics requires time and multiple didactic experiences.

A final distinction to be made is between precision public health applied to individuals and groups, such as families, and precision public health applied to populations. Professionals employ cascade screening to hunt down further family members who are at risk for infectious or genetic disease. Although, socially-minded authorities have also viewed it important to target geographic areas or populations based on basic healthcare needs, such as the need for nutritional supplementation, HIV therapy, or to address the problems of racial residential segregation [46,61]. Indeed, county of residence, with data obtained from the National Center for Health Statistics and population counts from the Census Bureau, may be the most telling indicator of mortality risk [62]. The collective approach to targeted health is only lightly if at all touched on by the PMI, but is critical to a complete precision public health approach to disease risk.

The *All of Us* Research Program stands at an equilibrium point between two wave fronts—precision medicine and precision public health. Its products will be used to develop new targeted therapies, and new predictive measures such as risk scores. It is an energetic lead for both, with more developments yet to come. Inclusion of the Precision Medicine Initiative in a public health course like HMP 517 lays the anchor on what needs to be taught and discussed. We are very pleased to have been able to incorporate this unique point in time capturing the start of a national precision medicine project into the course. The techniques that comprise precision medicine and precision public health stand to be strengthened by the PMI in the same way that the arsenal of useful genetic tests was boosted by the Human Genome Project. A broader vision for curriculum development includes early-stage disease pharmacogenomics [63], cascade screening [64], and targeted social interventions in the same vessel. Once the full chest of precision health tools is packed, it’s “anchors away!” for the next-generation of teaching precision public health.

## Figures and Tables

**Table 1:** Questions posed to students for the precision medicine and public health class exercise.

How can we assure that the large longitudinal cohort study will enlist volunteers representative of the demographic groups most relevant to addressing our major health problems? What kind of consent should be obtained for those participating in the cohort? How can we assure that the Initiative reduces and does not widen health disparities? How can we assure access to the drugs and therapies resulting from the Initiative by those for whom they will provide most benefit? How can we prevent the development and marketing of drugs targeting specific racial or ethnic groups from stigmatizing these groups? What privacy safeguards need to be built into the large cohort study? How might we achieve adequate involvement in the development and implementation of the Initiative by appropriate stakeholder groups? Who should have access to the database created by the large cohort study, and for what purposes? Should those receiving financial benefits from utilizing the database have an obligation to share the benefits for public health purposes? How can we prevent the increase in individualized health information from being used to discriminate in employment or insurance?

**Table 2:** Student responses in key precision medicine and public health class exercise areas.

Trust-Building Shareholder consultation before the implementation of a new treatment into clinical settings. (U.S. Centers for Disease Control and Prevention (CDC) Office of Public Health Genomics student representative); Researchers and participants being equally involved would establish trust. (National Society of Genetic Counselors student representative); A large number of people surveyed indicated they would be more likely to participate knowing their health information would be returned (National Society of Genetic Counselors student representative); The key to enrolling a diverse population is to work transparently with and gain the trust of communities and groups to utilize pre-existing networks. We must connect and engage with community and advocacy groups and then reach out to the public with the help of those groups and their websites and means of communication. We are also supportive of using social media such as Facebook and Twitter to increase diverse involvement (Genetic Alliance student representative); To build trust in the community, the PMI will need to work with community leaders, develop adequate privacy and data use agreements, and host focus groups to ensure vulnerable populations’ voices are heard in the development of recruitment and consent materials (National Urban League student representative).
Assuring access and equity Emphasis should be put into reaching out to members of disadvantaged communities who would not otherwise become informed about participating in the Precision Medicine Initiative (CDC Office of Public Health Genomics student representative); Both the design of new drug trials, and the 1M+ cohort must include individuals from underserved groups. … Expanding payment policies to cover new therapies that serve the underserved (Pharmaceutical Research and Manufacturers of America (PhRMA) student representative); New technologies must meet social needs. Problems rooted in poverty, racism, and other forms of inequality cannot be remedied by technology alone (Council for Responsible Genetics student representative); The development of new pharmacogenomics drugs and tests must not widen health disparities or support differential access to care. Current trends can be exacerbated by the pricing out of individuals from receiving appropriate health care needs (National Urban League student representative).
Ethics—Benefit sharing We believe that the data, particularly the genetic data from the one million person cohort, should be freely available to the public through online databases (American Society of Human Genetics student representative); There is an overwhelming need for resources to be made available to help individuals understand the meaning of genetic results to prevent harm (National Society of Genetic Counselors student representative); The dataset should be available for research purposes only, but available for all qualified academic, industry, and government workers (Pharmaceutical Research and Manufacturers of America (PhRMA) student representative); We strongly support open access and believe that all researchers should have access to this data. Furthermore, the public should have open access to published articles using this data (Genetic Alliance student representative); Determine how the genome interacts with exposures. The findings should focus on the individual and not on industry—the goal is what benefits people (Council for Responsible Genetics student representative); By reporting information on genetics in a manner in which the average person can understand, the project runners will not only be fulfilling a duty to the American people, but also facilitating the continuation of their research (Council for Responsible Genetics student representative).
Ethics—Consent and privacy What kind of consent should be obtained for those participating in the cohort? We should seek the broadest possible consent. Such a dataset does not fit the model of “here is exactly what I plan to do with the data”, but must be mined for insight. Consent should be established by a central IRB (institutional review board) committee composed of members representing all stakeholders (Pharmaceutical Research and Manufacturers of America (PhRMA) student representative); Anonymized data can be made available online to the public through VCF (variant call formatted) files like the 1000 Genomes Project with single nucleotide polymorphism (SNP) frequencies, genotypes and haplotypes. Non-genetic information—lifestyles and phenotypic data—can be made available by consent through appropriate controlled channels. NIH (the U.S. National Institutes of Health agency) has policy outlines for data sharing in genome-wide association studies. These need to be revamped and updated (American Society of Human Genetics student representative); They [participants] should also know who will have access to their data and whether it may be sold or transferred to outside groups, a concern which has become particularly important given the recent efforts to network biobanks and the sale by 23 and Me of genetic information to corporations. In addition, participants should be informed about whether their data will be “anonymized” (no identifying information available) or “de-identified” (no identifying information directly attached, but still accessible), and made aware that … it may still be possible to determine with some degree of certainty one’s identity from their genome. (Council for Responsible Genetics student representative).
